# Rural, Suburban, and Urban Differences in Chronic Pain and Coping Among Adults in North Carolina: 2018 Behavioral Risk Factor Surveillance System

**DOI:** 10.5888/pcd18.200352

**Published:** 2021-02-18

**Authors:** Ann P. Rafferty, Huabin Luo, Kathleen L. Egan, Ronny A. Bell, N. Ruth Gaskins Little, Satomi Imai

**Affiliations:** 1 Department of Public Health, Brody School of Medicine, East Carolina University, Greenville, North Carolina; 2 Department of Health Education and Promotion, College of Health and Human Performance, East Carolina University, Greenville, North Carolina

## Abstract

**Introduction:**

Our study aimed to examine the prevalence of chronic pain, its severity, its causes, and coping mechanisms that are used by North Carolina adults in rural, suburban, and urban areas.

**Methods:**

We analyzed data from the Behavioral Risk Factor Surveillance System’s first chronic pain module in 2018, representing 3,598 respondents. Self-reported chronic pain was defined as the affirmative response to the question, “Do you suffer from any type of chronic pain, that is, pain that occurs constantly or flares up often?” We computed prevalence of chronic pain and use of coping mechanisms by rural, suburban, or urban residential status. We used multiple logistic regression to assess the association between chronic pain and residential location, adjusting for demographic characteristics, employment, and health insurance.

**Results:**

In 2018, an estimated 27.5% (95% confidence interval [CI], 25.6%–29.3%) of North Carolina adults experienced chronic pain. Prevalence of chronic pain in rural areas (30.9%) and suburban areas (30.8%) was significantly higher, compared with urban areas (19.6%). Compared with urban residents with chronic pain, those with chronic pain in suburban areas (adjusted odds ratio [AOR], 0.44; 95% CI, 0.26–0.76) and in rural areas (AOR, 0.39; 95% CI, 0.24–0.65) were less likely to use nonmedication therapies (eg, acupuncture, physical therapy, yoga) and were less likely to use 3 or more types of chronic pain treatment (suburban AOR, 0.47; 95% CI, 0.25–0.88; rural AOR, 0.53; 95% CI, 0.29–0.95).

**Conclusion:**

Our results indicate that persons living in rural and suburban areas may be more likely to have chronic pain and less likely to use nonmedication treatments than those in urban areas.

**Summary**

**What is already known about this topic?**

Chronic pain is more prevalent among US adults living in rural areas than in urban areas; however, the literature on prevalence and treatment differences is limited.

**What is added by this report?**

Using a representative sample, we showed that North Carolina adults living in suburban and in rural areas are 60% more likely to have chronic pain than those in urban areas. Additionally, adults with chronic pain in suburban and rural areas were less likely to use nonmedication treatments and less likely to use 3 or more types of treatments compared with adults in urban areas.

**What are the implications for public health practice?**

Public health interventions are needed to increase availability of nonmedication treatments for chronic pain in suburban and rural areas.

## Introduction

Chronic pain is a significant public health problem that can lead to disability, opioid addiction, depression, and lower quality of life (1). Annually, 20.4% of US adults are affected by chronic pain, representing one of the most common reasons adults seek medical care (2,3). Medical costs directly related to chronic pain climbed to $635 billion in 2012 (4). Causes are multifaceted, including injury, underlying medical conditions, medical procedures, and inflammation (5). Pharmacologic and nonpharmacologic treatment options are available, depending on the cause of pain (5).

Chronic pain and its consequences are not equally distributed in the population. Higher prevalence is reported more frequently among older adult women than other groups, those previously but not currently employed, those experiencing poverty, and those having public health insurance (2). Pain severity is greater for non-Hispanic Black and Hispanic people than for people of other racial/ethnic groups, greater for Medicaid recipients than nonrecipients, greater for adults with chronic disease than those without, greater for those experiencing psychological distress, and greater for those with lower educational levels than those with at least a high school diploma (6). Residents of rural areas are more likely to experience chronic pain than residents of urban areas (2,7). Rural residents experiencing chronic pain in the lower back are more likely to have functional limitations and less likely to receive specialty care compared with their urban counterparts, (8).

Following release of the 2011 Institute of Medicine report, Relieving Pain in America (1), the Interagency Pain Research Coordinating Committee created the National Pain Strategy (5), a comprehensive plan to address pain prevention and management, particularly among populations at increased risk for chronic pain (5). One recommendation was to increase US population-based research about chronic pain, including the prevalence of and treatments for chronic pain.

We sought to expand the body of population-based knowledge about chronic pain, as recommended by the National Pain Strategy*,* by examining how the prevalence of self-reported chronic pain, its severity, causes, and coping mechanisms vary among rural, suburban, and urban residents of North Carolina.

## Methods

We analyzed data from the 2018 North Carolina Behavioral Risk Factor Surveillance System (BRFSS), which comprises annual, state-level, telephone surveys of adults that use probability samples of all households with landline or cells phones in North Carolina (9). The BRFSS is coordinated by the Centers for Disease Control and Prevention and collects self-reported information on health behaviors and preventive health practices related to the leading causes of death and disability. Participating states have the option to include additional questions to the standard BRFSS. In 2018, for the first time, the North Carolina BRFSS asked questions about chronic pain, based on a survey module included in the 2007 Kansas BRFSS (10), to a representative sample of 3,598 adults. The response rate for the 2018 North Carolina BRFSS was 43.5%, compared with the median response rate of 49.9% and a range of 38.8% to 67.2% for all participating states and territories (11).

### Outcome variables

Responses to 2 questions were used to define the chronic pain analysis variable: 1) “Do you suffer from any type of chronic pain, that is, pain that occurs constantly or flares up often?” and 2) “About how often do you experience this pain? Would you say . . . it’s constant, always there, at least once a day, at least once a week, at least once a month, or less often?” Respondents were classified as having chronic pain if they responded yes to the first question and reported that they experienced this pain at least once a week (n = 1,067). Respondents were classified as not having chronic pain if they responded no to the first question (n = 2,437), or yes to the first question but reported pain frequency as less than once a week (n = 94). A total of 2,531 respondents were classified as not having chronic pain. The decision to restrict the definition of chronic pain to those who experienced pain at least once a week was based on the definition of chronic pain used in the National Pain Strategy as pain that occurs on at least every other day for 6 months or more (5).

#### Coping mechanisms

Respondents were asked whether they did anything to cope with their chronic pain. Those who answered yes were asked a series of 9 yes or no questions about specific treatment types. That is, did the respondent use: 1) over-the-counter medication, such as ibuprofen or aspirin; 2) a prescription anti-inflammatory drug, such as Celebrex; 3) a prescription narcotic pain reliever, such as Percocet or Vicodin; 4) some other prescription drug; 5) a nonmedication pain therapy, such as acupuncture, physical therapy, or yoga; 6) alcohol; 7) marijuana; 8) a street drug other than marijuana; or 9) something else. We created 3 treatment-related analysis variables. First, respondents were classified as using a nonnarcotic prescription medication if they answered yes to using a prescription anti-inflammatory or to using some other prescription medication, and answered no to using a prescription narcotic. Second, substance use was defined as a yes response to using alcohol, marijuana, or a street drug other than marijuana. Third, number of treatment types was summed across over-the-counter medications, any prescription medication, nonmedication therapy, substance such as alcohol, marijuana, or a street drug, and something else. This sum was recoded to a 2-level variable (≤2 vs ≥3 treatment types).

#### Severity and main cause of chronic pain

Severity of chronic pain was assessed by the question, “On a scale of 0 to 10, where 0 means no pain at all and 10 means the worst pain you can imagine, how severe has your pain usually been over the past 3 months?” Following the definition used by the North Carolina BRFSS (12), we classified this pain scale on 3 score levels: mild, 1 to 5, moderate, 6 to 7, and severe, 8 to 10. Cause of chronic pain was assessed by the question, “What is the main cause of your chronic pain?” Ten response options were given in the questionnaire: migraine; cancer; arthritis; shingles; sciatica, slipped disk or spondylosis; diabetes; muscle pain; accident or injury; neuropathic pain; and other. Only 1 response was allowed per respondent. An additional cause of pain category (hip, knee, foot, or other joint pain) was coded after data collection, based on other specified responses. Causes of pain that received 16 or fewer responses were combined into the other category (ie, migraine [n = 16], cancer [n = 10], shingles [n = 2], diabetes [n = 15]). We created 1 dichotomous variable for each main cause of pain.

#### Independent variable

The independent variable for this analysis was residential status (urban, suburban, rural). We followed the North Carolina Rural Center’s county-based classification (13) used by the North Carolina BRFSS (14). Rural counties were defined as having an average population density of 250 people or less per square mile; suburban counties were defined as having an average population density between 250 and 750 people per square mile; and urban counties were those with an average population density more than 750 people per square mile.

#### Covariates

The demographic characteristics examined were: age (18–34, 35–49, 50–64, ≥65 years); sex (male, female); race/ethnicity (non-Hispanic White, non-Hispanic Black, Hispanic, other); education (less than high school graduate, high school graduate or GED, some college or technical school, college graduate), annual household income (<$20,000, $20,000–34,999, $35,000–49,999, $50,000–74,999, ≥$75,000, missing); and current employment status (employed, out of work, not working, unable to work). Health insurance status (yes, no) was also included as a covariate since its presence or absence would likely influence the type of pain relief sought, and it was defined by the question, “Do you have any kind of health care coverage, including health insurance, prepaid plans such as HMOs, government plans such as Medicare, or Indian Health Service?”

### Statistical analysis

Data were analyzed by using SAS version 9.4 (SAS Institute). The percentage distributions of sample characteristics among those with and without chronic pain were calculated. The prevalence of chronic pain, types of treatment, and the frequency, severity, and main cause of chronic pain were calculated by residential status. Rao-Scott χ^2^ statistics were used to test bivariate relationships. We used multiple logistic regression to assess the associations between having chronic pain and residential location, adjusting for the covariates. Survey procedures were used to account for sampling design and weighting factors. All results, except for sample sizes, were weighted.

## Results

### Chronic pain

In 2018, the prevalence of chronic pain among North Carolina adults was estimated at 27.5% (95% confidence interval [CI], 25.6%–29.3%), with significant variation by residential status (*P* < .001). An estimated 30.9% (95% CI, 28.2%–33.6%) of adults in rural counties, 30.8% (95% CI, 26.9%–34.6%) in suburban counties, and 19.6% (95% CI, 16.5%–22.7%; *P* < .001) of adults in urban counties had chronic pain.

A higher proportion of North Carolina adults with chronic pain lived in rural counties, and a lower proportion lived in urban counties, compared with the distribution of those without chronic pain (Table 1). The proportion of North Carolina adults with chronic pain was higher for women than men, for those aged 50 years or older than those who were younger, for non-Hispanic White than those of other races/ethnicities, for those living in households with annual incomes less than $20,000 than households with higher incomes, for those unable to work than those of other employment status, and for those with less than a college degree. Compared with urban residents, the adjusted odds of chronic pain were 36% higher among rural residents (adjusted odds ratio [AOR], 1.36; 95% CI, 1.05–1.76) and 51% higher among suburban residents (AOR, 1.51; 95% CI, 1.12–2.03) (Table 2).

**Table 1:** Sample Characteristics by Presence of Chronic Pain, Percentage Distribution and Unweighted Sample Size, North Carolina Behavioral Risk Factor Surveillance System, 2018

Characteristics	Has Chronic Pain (n = 1,067), n (%)	No Chronic Pain (n = 2,531), n (%)	*P* Value^b^
**Residential status^a^**			
Urban	186 (21.4)	678 (33.3)	<.001
Suburban	259 (30.3)	575 (25.8)	
Rural	622 (48.3)	1,278 (40.9)	
**Age, y**			
18–34	102 (14.3)	519 (29.4)	<.001
35–49	210 (23.7)	537 (23.3)	
50–64	360 (34.7)	655 (25.3)	
≥65	380 (27.4)	783 (22.0)	
**Sex**			
Male	423 (39.2)	1,199 (49.0)	<.001
Female	644 (60.8)	1,330 (51.0)	
**Race/ethnicity**			
White, non-Hispanic	765 (72.8)	1,633 (64.1)	<.001
Black, non-Hispanic	182 (17.8)	505 (22.2)	
Hispanic	40 (3.7)	235 (8.6)	
Other	67 (5.7)	127 (5.0)	
**Education**			
Less than high school diploma	130 (15.3)	284 (14.1)	<.001
High school diploma	292 (26.6)	595 (25.6)	
Some college	365 (39.6)	706 (31.9)	
College degree	280 (18.5)	938 (28.4)	
**Annual household income, $**			
<20,000	249 (21.5)	330 (12.4)	<.001
20,000–34,999	205 (18.5)	413 (15.2)	
35,000–49,999	125 (12.3)	298 (10.9)	
50,000–74,999	127 (12.7)	350 (14.1)	
≥75,000	172 (17.6)	645 (27.3)	
Missing	189 (17.4)	495 (20.2)	
**Employment status**			
Employed	370 (40.5)	1,399 (60.8)	<.001
Out of work	37 (4.0)	74 (3.6)	
Not working^c^	392 (30.6)	(32.1) 943	
Unable to work^d^	263 (24.8)	(3.5) 101	
**Health insurance**			
Has health insurance	950 (89.0)	2,164 (84.3)	.04
No health insurance	116 (11.0)	362 (15.7)	

Abbreviations: % = weighted percentage; n = unweighted sample size.

a North Carolina Rural Center’s county-based classification (13).

b Rao-Scott χ^2^.

c Includes the response categories “out of work for 1 year or more” and “out of work for less than 1 year.”

d Includes the response categories “homemaker” and “student.”

**Table 2:** Unadjusted and Adjusted Odds Ratios for Chronic Pain by Urban, Suburban, and Rural Status and Covariates, North Carolina Behavioral Risk Factor Surveillance System, 2018 (N = 3,598)

Characteristics	Unadjusted Odds Ratio (95% CI)	*P* Value^a^	Adjusted Odds Ratio^b^ (95% CI)	*P* Value^a^
**Residential status**				
Urban	1 [Reference]	<.001	1 [Reference]	.02
Suburban	1.79 (1.36–2.36)		1.51 (1.12–2.03)	
Rural	1.86 (1.46–2.36)		1.36 (1.05–1.76)	
**Age, y**				
18–34	1 [Reference]	<.001	1 [Reference]	<.001
35–49	2.19 (1.59–3.02)		2.13 (1.51–3.00)	
50–64	2.92 (2.16–3.95)		2.09 (1.50–2.89)	
≥65	2.69 (1.99–3.64)		2.09 (1.45–3.00)	
**Sex**				
Male	1 [Reference]	<.001	1 [Reference]	.01
Female	1.48 (1.23–1.79)		1.40 (1.14–1.72)	
**Race-ethnicity**				
White, non-Hispanic	1 [Reference]	<.001	1 [Reference]	<.001
Black, non-Hispanic	0.71 (0.56–0.91)		0.62 (0.48–0.82)	
Hispanic	0.35 (0.22–0.55)		0.52 (0.31–0.88)	
Other	1.03 (0.67–1.59)		1.13 (0.71–1.80)	
**Education**				
Less than high school graduate	1.68 (1.22–2.33)	<.001	1.11 (0.74–1.68)	.001
High school graduate	1.55 (1.21–1.99)		1.21 (0.91–1.61)	
Some college	1.88 (1.49–2.37)		1.68 (1.28–2.20)	
College graduate	1 [Reference]		1 [Reference]	
**Annual household income, $**				
<20,000	2.62 (1.94–3.55)	<.001	1.55 (1.05–2.28)	.01
20,000–34,999	1.85 (1.36–2.52)		1.65 (1.16–2.35)	
35,000–49,999	1.75 (1.24–2.45)		1.62 (1.12–2.35)	
50,000–74,999	1.42 (1.00–2.01)		1.27 (0.88–1.82)	
≥75,000	1 [Reference]		1 [Reference]	
Missing	1.30 (0.95–1.78)		1.01 (0.70–1.45)	
**Employment status**				
Employed	1 [Reference]	<.001	1 [Reference]	<.001
Out of work	1.73 (1.04–2.89)		1.92 (1.11–3.31)	
Not working^c^	1.40 (1.13–1.74)		1.07 (0.80–1.44)	
Unable to work^d^	10.58 (7.47–15.00)		7.77 (5.32–11.36)	
**Health insurance**				
Has health insurance	1.52 (1.14–2.03)	.01	1.27 (0.88–1.84)	0.20
Has no health insurance	1 [Reference]		1 [Reference]	

a *P* values determined by *F* tests.

b Adjusted for age, sex, race/ethnicity, education, household income, employment status, and health insurance.

c Includes the response categories “out of work for 1 year or more” and “out of work for less than 1 year.”

d Includes the response categories “homemaker” and “student.”

#### Coping with chronic pain

Of North Carolinians reporting chronic pain (n = 1,067), the majority (92.5%) visited or consulted a doctor or other health professional about their pain, and this did not differ by residential status in adjusted analyses (*P* = .47) (Table 3). Nearly 20% of respondents reported that they did nothing to cope with their pain, although 57.0% used an over-the-counter medication; 18.4% used a narcotic prescription medication; 41.2% used a nonnarcotic prescription medication; 25.4% used a nonmedication pain therapy; 6.3% used alcohol, marijuana, or street drugs; and 12.8% used something else to cope with their pain. Nonmedication therapies were the only treatment type that varied significantly by residential status (urban 42.6%, suburban 22.3%, rural 19.8%, *P* < .001). The adjusted odds of using nonmedication pain therapy were about 60% lower in suburban counties (AOR, 0.44; 95% CI, 0.26–0.76) and rural counties (AOR, 0.39; 95% CI, 0.24–0.65) compared with urban counties. Over 16% of those with chronic pain used 3 or more different types of treatments, and this varied by residential status with approximately 50% lower adjusted odds of using 3 or more treatments in suburban (AOR, 0.47; 95% CI, 0.25–0.88) and rural areas (AOR, 0.53; 95% CI, 0.29–0.95) compared with urban areas. We present the prevalence of coping mechanisms by residential status (Figure).

**Table 3:** Prevalence of Coping Mechanisms and Adjusted Odds Ratios by Residential Urban, Suburban, and Rural Status, North Carolina Behavioral Risk Factor Surveillance System 2018 (N = 1,067)

Coping Mechanism	Total, % (95% CI)	Residential Urban-Rural Status			
		Urban	Suburban AOR^a^ (95% CI)	Rural AOR (95% CI)	*P* Value^b^
Visited doctor about pain	92.5	1 [Reference]	0.58 (0.22–1.50)	0.80 (0.32–1.97)	.47
Doing nothing to cope with pain	19.3 (16.1–22.4)	1 [Reference]	1.69 (0.89–3.20)	1.53 (0.84–2.77)	.25
Using over-the-counter medication (eg, ibuprofen or aspirin)	57.0 (53.2–60.9)	1 [Reference]	0.77 (0.47–1.26)	0.83 (0.53–1.31)	.57
Using prescription narcotics (eg, Percocet, Vicodin)	18.4 (15.5–21.4)	1 [Reference]	1.44 (0.75–2.77)	1.22 (0.68–2.19)	.54
Prescription nonnarcotic medication	41.2 (37.3–45.2)	1 [Reference]	0.79 (0.48–1.30)	0.78 (0.49–1.23)	.54
Nonmedication pain therapy (eg, acupuncture, physical therapy, yoga)	25.4 (21.9–29.0)	1 [Reference]	0.44 (0.26–0.76)	0.39 (0.24–0.65)	.001
Alcohol, marijuana, or street drug	6.3 (4.4–8.2)	1 [Reference]	1.43 (0.49–4.21)	1.24 (0.43–3.60)	.80
Other	12.8 (10.0–15.5)	1 [Reference]	1.54 (0.78–3.04)	0.93 (0.50–1.74)	.20
Using ≥3 types of coping mechanisms^c^	16.6 (13.5–19.7)	1 [Reference]	0.47 (0.25–0.88)	0.53 (0.29–0.95)	.04

Abbreviations: AOR, adjusted odds ratio; CI, confidence interval.

a Adjusted for age, race/ethnicity, education, household income, employment status, and health insurance.

b *P* values determined by *F* test.

c Includes over-the-counter medications, prescription medications, nonmedication pain therapy, a substance such as alcohol, marijuana or street drugs, or something else.

**Figure:**
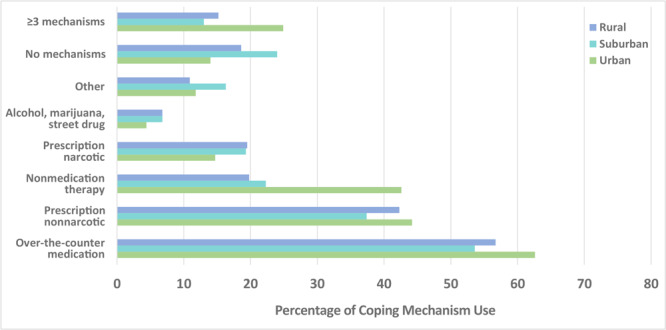
Prevalence of coping mechanism use for chronic pain among North Carolina adults and among those living in urban, suburban, and rural areas, North Carolina Behavioral Risk Factor Surveillance System, 2018.

#### Frequency, severity, and main cause of chronic pain

A majority (54.0%; 95% CI, 50.1%–57.9%) of respondents with chronic pain (n = 1,057) reported that the pain was constant, 30.8% (95% CI, 27.2%–34.4%) reported experiencing pain at least once daily, and 15.2% (95% CI, 12.4%–18.1%) experienced pain once a week. Almost one-half were classified as having mild pain (45.4%; 95% CI, 41.5%–49.3%), 22.4% (95% CI, 19.2%–25.5%) had moderate pain, and 32.3% (95% CI, 28.5%–36.0%) had severe pain. Neither pain frequency (*P* = .06) nor severity (*P* = .31) varied significantly by residential status. The most frequently reported main cause of chronic pain was arthritis (32.2%; 95% CI, 28.7%–35.7%), followed by sciatica, slipped disk, or spondylosis (17.6%; 95% CI, 14.9%–20.3%); accident or injury (11.6%; 95% CI, 9.0%–14.1%); muscle pain (6.3%; 95% CI, 4.5%–8.1%); neuropathic pain (5.3%; 95% CI, 3.4%–7.2%); and hip, knee, foot, or other joint pain (4.9%; 95% CI, 3.0%–6.8%). Nearly 17% (16.6%; 95% CI, 13.4%–19.8%) reported another primary cause of their pain, and 5.5% (95% CI, 3.5%–7.6%) did not know the cause of their pain. The proportions who reported neuropathic and joint pain as their main cause of chronic pain differed significantly by residential status, although other causes did not. In rural areas, 8.2% of those with chronic pain had neuropathic pain, although 2.0% in urban areas and 2.9% in suburban areas had neuropathic pain (*P* = .006). The adjusted odds of neuropathic pain were 3.5 times higher in rural areas compared with urban areas (AOR, 3.57; 95% CI, 1.02–12.46). Joint pain was a less frequent cause of chronic pain among suburban residents (2.0%) and rural residents (3.8%), compared with urban residents (11.3%, *P *< .001), with adjusted odds of joint pain being over 70% lower in suburban (AOR, 0.15; 95% CI, 0.05–0.50) and rural (AOR, 0.27; 95% CI, 0.11–0.71) areas compared with urban areas.

## Discussion

Chronic pain is a public health issue that greatly affects a large segment of the adult population and contributes to high morbidity, disability, and health care costs in the United States (3,4,15). Findings from our study add to the limited body of literature on chronic pain. First, the prevalence of self-reported chronic pain was nearly 60% higher among North Carolina adults in suburban and rural communities compared to those in urban communities. About 1 in 3 adults in suburban and rural communities reported chronic pain, which represents a substantial segment of the population. Second, suburban and rural residents were significantly less likely to report nonmedication treatments for chronic pain, such as physical therapy, acupuncture, or yoga. Third, suburban and rural residents were less likely to report using multiple treatments (≥3) for chronic pain. The latter 2 points speak to disparities in chronic pain treatment for residents in less populated communities.

The 2018 North Carolina BRFSS data showed that 27.5% of North Carolina adults experience chronic pain, which is slightly higher than the 2016 national estimate of 20.4% (2), and within the range of reported prevalence estimates from 11% to 40% (5). Our results on chronic pain prevalence by urban–rural residential status concurred with those from other researchers who have found that rural populations are especially susceptible to the impact of chronic pain (2,7,8). North Carolina has a large rural population and therefore may be particularly vulnerable to this condition (13). Chronic pain may be more common in rural communities as a result of the higher rates of employment in jobs that might increase risk for chronic pain, such as agriculture and manufacturing (16). Age of the population on average is older in rural counties than urban counties in the United States (17), which might be a contributing factor to the higher prevalence of chronic pain found in rural areas. Our adjusted analysis, however, confirmed higher odds of chronic pain in rural and suburban areas compared with urban areas even after adjusting for age and other covariates.

Knowing the cause of pain can inform decisions about pain treatment: pharmacologic, nonpharmacologic, or a combination of both (5). Our results indicate that arthritis was the most common cause of chronic pain in North Carolina, with 32.2% of those with chronic pain reporting it as the main cause of their pain. In the literature, lower back pain is often cited as causing the greatest global burden (18). Comparisons among study results are difficult to make because of the different questions and methodologies used. In the North Carolina BRFSS question, the focus was on underlying cause rather than the area of the body experiencing pain. We also found that neuropathic chronic pain was higher in suburban and rural areas compared with urban areas. A possible explanation for this finding is that the prevalence of diabetes is higher in rural North Carolina compared with urban areas (19), and diabetes can result in neuropathy (20).

In 2018, more than 1,700 unintended opioid-related deaths and approximately 6,770 emergency department visits occurred among North Carolina residents as a result of opioid overdose (21). The recent opioid epidemic has especially devastated rural communities across the United States (22), highlighting the need to improve understanding of nonopioid treatment options for chronic pain in rural America. Although we did not find a significant difference in use of pharmacologic treatments (eg, over the counter and opioid and nonopioid prescription medication), suburban and rural residents were significantly less likely to report nonmedication treatments for chronic pain, such as physical therapy, acupuncture, or yoga. Furthermore, suburban and rural residents were less likely to report using 3 or more treatment types, which is concerning given that multimodal, interdisciplinary treatments for chronic pain are recommended over single-modal treatments that often fail (5). The Institute of Medicine recommends a comprehensive approach to treatment of chronic pain that includes the use of nonmedication approaches, such as psychological therapies, rehabilitative and physical therapy, and complementary and alternative medicine (eg, acupuncture) (1).

Our findings suggest that rural residents in North Carolina are not receiving or engaging in the recommended comprehensive care for their chronic pain. Therefore, addressing geographic disparities in nonmedication and multimodal interdisciplinary treatment of chronic pain by using multiple approaches is critical, including increasing the accessibility of nonmedication treatments in suburban and rural areas, coverage of nonmedication treatment by health insurance programs, and specific interventions for individuals with chronic pain. Regarding accessibility of nonmedication treatments, rural areas often have fewer medical specialists and rely on primary care providers (PCP) for the treatment of pain (23). Because PCPs typically have minimal education on the treatment of chronic pain (24), improved training of PCPs in pain management and referring chronic pain patients to other practitioners for nonmedication treatments may be promising options to address geographic disparities. Physical therapy is a medical treatment that can be billed to insurance, although acupuncture and yoga might not be covered (25). Understanding if disparities exist in the use of physical therapy would provide an avenue for intervention covered by health insurance, especially because health insurance was not significantly associated with chronic pain in adjusted analysis. Finally, comprehensive chronic pain self-management programs implemented in suburban and rural areas by nurses or other midlevel health professionals may reduce geographic disparities of chronic pain treatment (23).

This study has limitations. First, data are self-reported, which would increase the likelihood of response bias, and the BRFSS only interviews community-dwelling adults with access to a telephone, which may contribute to a lack of generalizability to certain populations. Second, the definition of chronic pain used in our analysis was limited by the survey questions asked and may have resulted in an overestimate of the prevalence of chronic pain compared with the National Pain Strategy definition. Third, nonmedication therapy use was assessed by 1 question that asked about physical therapy, acupuncture, and yoga together, so that use of each of these treatments cannot be distinguished from one another in the response, and this question leaves out other recommended nonmedication treatments (eg, psychological therapies [1]). Fourth, responses to the cause of chronic pain questions may have been influenced by the set of precoded response categories and may not represent a full understanding of the cause of pain, and this question did not allow for the specification of multiple causes of pain. Fifth, we were not able to fully assess the types of treatment used by nearly 13% of respondents with chronic pain, who reported that they were using “something else” to treat their pain. Finally, because this was a cross-sectional survey, we were not able to assess the long-term consequences of chronic pain or to investigate causation.

Despite these limitations, this analysis has strengths, including the large sample size, which allowed for subgroup analyses, and the use of a standardized survey instrument for assessing chronic pain, which allowed us to look at both the characteristics of chronic pain and coping strategies for this population. Thus, our findings add to the limited body of knowledge on the impact of chronic pain in rural, suburban, and urban communities.

This study indicates that North Carolina adults living in rural and suburban areas were more likely than adults living in urban areas to report experiencing chronic pain and less likely to report accessing nonmedication treatments for their condition. Further research is needed to better elucidate this phenomenon, especially the use of nonmedication treatment and coping mechanisms in rural areas, to provide policy makers with evidence to enhance pain treatment resources in these communities and to address chronic pain disparities.
